# Tripled Readout Slices in Multi Time-Point pCASL Using Multiband Look-Locker EPI

**DOI:** 10.1371/journal.pone.0141108

**Published:** 2015-11-06

**Authors:** Ke Zhang, Seong Dae Yun, N. Jon Shah

**Affiliations:** 1 Institute of Neuroscience and Medicine– 4, Medical Imaging Physics, Forschungszentrum Jülich, Jülich, Germany; 2 Department of Neurology, Faculty of Medicine, JARA, RWTH Aachen University, Aachen, Germany; University Medical Center (UMC) Utrecht, NETHERLANDS

## Abstract

Multi time-point pseudo-continuous arterial spin labelling (pCASL) with a Look-Locker EPI readout can sample the signal curve of blood kinetics at multiple time points after the labelling pulse. However, due to signal relaxation of labelled blood, the number of readout slices is limited. The aim of this study is to employ a multiband excitation technique to triple the number of readout slices in multi time-point pCASL. The multiband technique, along with 2-fold in-plane parallel imaging, was incorporated into the Look-Locker EPI for the multi time-point sampling of blood kinetic behaviour following the pCASL labelling scheme. The performance evaluation of the multiband and the single-band techniques were performed on four healthy subjects using a 32-channel head RF coil at 3T. Quantitative perfusion maps were analysed using a combination of labelling with and without flow suppression gradients. The perfusion maps provided by the multiband accelerated multi time-point pCASL were in good agreement with the conventional single-band technique. Multiband acceleration caused SNR loss but offered quantitative perfusion maps in 6.23 min with 18 slices compared with 6 slices within the same time period for the single-band method. As conclusion, the multiband technique can successfully triple the number of readout slices while achieving comparable perfusion data in the same measurement time as the conventional single-band readout.

## Introduction

Arterial spin labelling (ASL) employs no exogenous contrast agents, or radioactive substances and is a low-cost alternative to determine perfusion with PET- or contrast agent-based MRI [1]. The concept of ASL is to magnetically label spins from the arterial blood by pulsed inversion in a tagged image and subtract it from the control image that has no spin labelling. Among different ASL tagging techniques, pseudo-continuous arterial spin labelling (pCASL), with a long RF train of more than 1s as a labelling scheme, can provide high signal-to-noise ratio (SNR) perfusion weighting [2, 3]. Following the labelling pulse, a post-labelling delay (PLD) is inserted before image acquisition to allow the labelled blood to reach the tissue. However, due to variance in the blood arrival time (BAT) for different regions of the brain, the choice of a suitable PLD is difficult. One possible option to solve this problem is to insert a delay larger than BAT, which will provide a correct measurement of CBF with just a single PLD.

To quantitatively measure cerebral blood flow (CBF) with ASL, multiple readouts with different PLDs are preferable in the case of variable BATs [4]. One approach for sampling the tracer kinetics curve with increasing PLDs is to use Look-Locker (LL) imaging following the labelling scheme [5–9]. Instead of catching the perfusion signal after one inversion delay, LL-EPI uses a series of low flip-angle EPI readout modules to monitor the inversion recovery of the labelled spins following the inversion pulse. Look-Locker ASL can measure perfusion in a more temporally-efficient way than the single readout pulse used in conventional ASL [5] and has the advantage that all PLD are acquired at exactly the same time. Usually, two-dimensional (2D) multislice EPI acquisitions are sequentially obtained at multiple time points with the subsequent fitting of the kinetic model to the data in order to quantify CBF. During the readout period, the longitudinal magnetization of the tagged spins relaxes. In order to acquire the kinetic behaviour of the tagged spins quickly before they fully relax, the number of slices and the readout time points have to be reduced. Typically, 7 slices and 13 time points are required in LL-EPI based QUASAR (quantitative STAR labelling of arterial regions) within a total measurement time of 6 minutes [9, 10]. To have whole brain quantitative CBF, clearly a larger number of readout slices are required. Multiple concatenations can help to cover more slices, but this increases measurement time.

Recently, simultaneous multi-slice (SMS) imaging using multiband (MB) excitation has been presented to accelerate volumetric acquisition [11–13]. Here, a number of slices, *m*, can be simultaneously excited using a MB composite RF pulse such that the acquisition time can be shorted by a factor of *m*, or alternatively, brain coverage can be increased by the same factor whilst the acquisition time is maintained. Moreover, to avoid high g-factor penalties in the slice separation of SMS, the blipped-CAIPI (blipped-controlled aliasing in parallel imaging) acquisition scheme has been presented [12]. By applying small blipped gradients along the slice selection direction during read gradient switching, a desired object shift between the simultaneously excited slices can be created along the phase encoding direction [13]. This shift reduces the high g-factor penalties and effectively reduces the noise amplification in the reconstructed images. The advantages of the MB technique have already been demonstrated for functional MRI studies and diffusion-based fibre tractography [11, 13–15]. Applications of the MB technique have been demonstrated in the single-shot pulsed ASL [16, 17]. However, none of them have shown the advantage of MB method in multi time-point ASL studies.

In this work, we propose to use MB EPI in multi time-point pCASL (MEM-pCASL) to triple the number of readout slices for whole brain quantitative perfusion measurements within the same measurement time as with the single-band (SB) technique. After the combination of image acquisition with and without flow crusher gradients, macrovascular and tissue signals can be separated out and quantitative CBF analysed. The MEM-pCASL sequence labels blood spin using the pCASL labelling pulses and acquires multiple inversion time information using an LL-EPI readout adapted to include the MB technique.

## Methods

The performance of the MB and SB excitation methods was evaluated using data from four young (24~29 years) healthy subjects using a 32-channel head RF coil on a 3T Trio Siemens scanner (Siemens Healthcare, Erlangen, Germany). Written, informed consent was obtained from all subjects and the study was approved by the Ethics Committee of the Medicine Faculty of the Rheinisch-Westfälischen Technischen Hochschule Aachen (RWTH Aachen University). The study was conducted according to the Declaration of Helsinki.

### Multiband Excitation

**Fig 1:**
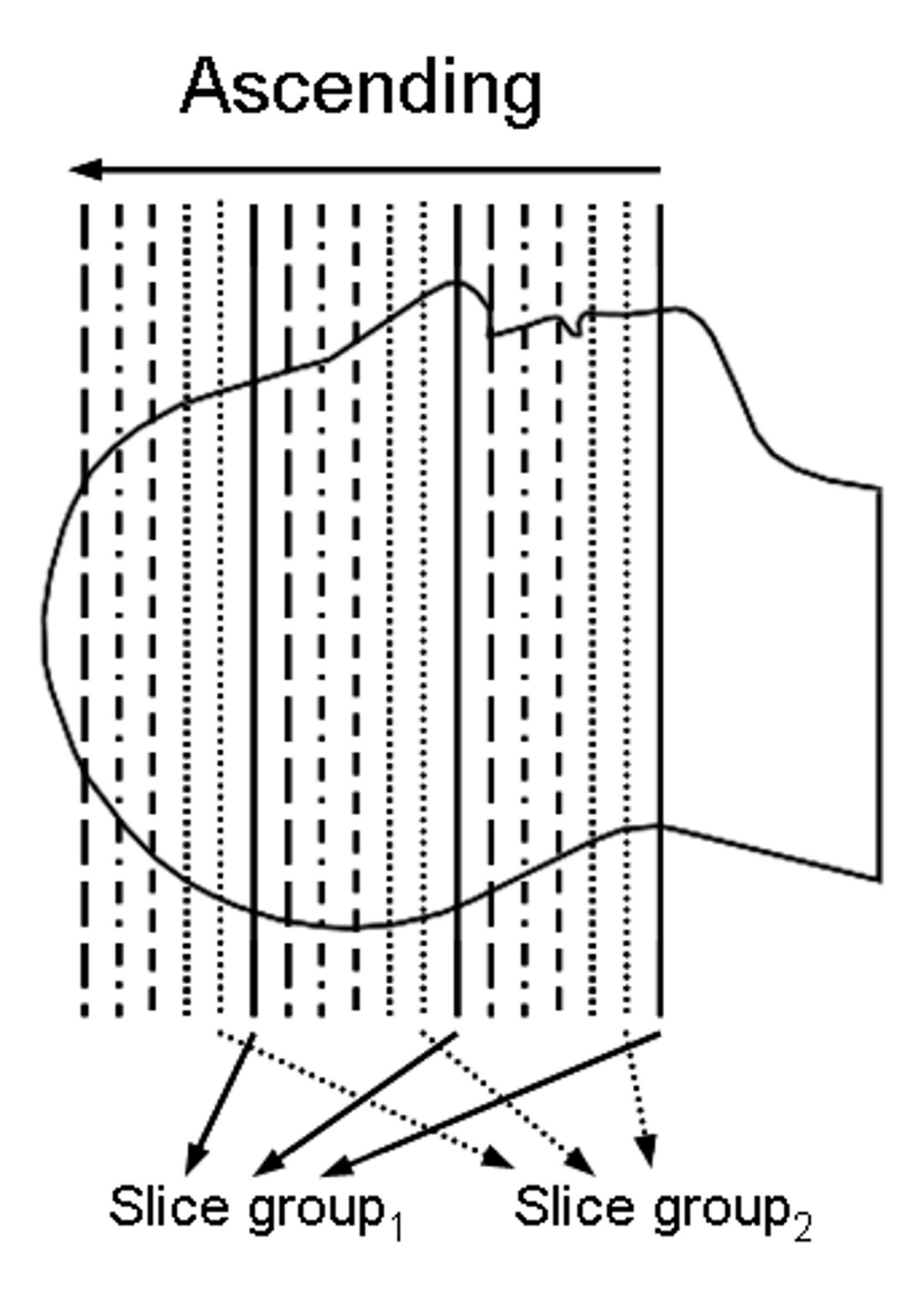
Schematic representation of the slice groups selected using MB RF pulses. The slice groups were recorded in the ascending direction. Three slices, shown in the same line style, are selected simultaneously by one MB RF pulse.

The MB technique achieves acceleration across the slice loop by exciting a number of slices simultaneously using multiband RF pulses per TR loop. With a linear combination of frequency-shifted pulses, multiple bands in the frequency domain of the RF are determined. The multiband RF produces multiple clones of the original single band profile, each of which has a symmetric frequency offset [18]; in the illustration shown in Fig 1, three slices are simultaneously excited. For the application of multi time-point pCASL, six slice groups were recorded in ascending order. Signal separation is performed based on the principles of parallel imaging reconstruction using distinct sensitivity profiles at each different slice position.

### Blipped-CAIPI

The MB data were reconstructed using the 'Slice-GRAPPA' algorithm as suggested by Setsompop et al. [15]. The fundamental elements to separate the individual slices from the overlapped signals are distinct sensitivity profiles of the 32 receivers at each different slice position. However, the fact that the sensitivity profiles in the overlapped area show very little difference between the simultaneously excited slices, incurring a high g-factor penalty and reconstructed images of potentially poor quality. In order to reduce this penalty, the approach of blipped-CAIPI was also used in this work. Image reconstruction after the application of MB technique was performed using Matlab (Mathworks Inc., Natick, MA, USA). A g-factor map was calculated after the application of MB data reconstruction; the effect of g-factor induced from the in-plane parallel acceleration is not shown since the in-plane acceleration was employed in both SB and MB imaging.

### ASL Experiments

In order to measure the local arterial input function (AIF), both crushed (c = 8cm/s) and non-crushed data were acquired in 7 cycles with crusher gradients in different directions: [(+x,+y,+z);(−x,+y,+z);none;(+x,−y,+z);(−x,−y,+z);none;none] [10]. These crusher gradients suppress the macrovascular signals from blood flowing at above 8 cm/s. To gain a fully-recovered signal, control-label pairs were acquired in an interleaved manner. Specific sequence parameters were as follows: labelling train = 1s; interpulse timing of the pCASL RF-train = 960 μs; flip angle = 35°; repetition time = 4500ms; echo time = 27ms; ΔPLD = 270ms; PLD_1_ = 50 ms; parallel imaging acceleration factor = 2; bandwidth = 1302 Hz/Px; slice acquisition order: ascending; voxel size = 3.4×3.4×6 mm^3^; and thickness of pre-saturation = 130 mm. The in-plane matrix size was 64×64 and the number of slice/slices gap of the acquisitions with an MB factor of 3 were 18/1.2 mm and for the SB acquisitions were 6/12 mm. The total measurement time for 42 control-label pairs containing 7 crush cycles was 6.23 min for both the MB and SB readout methods.

### Data Processing

The reconstructed images were pre-processed using the realignment, re-slicing and spatial smoothing (a Gaussian kernel of 4mm FWHM) steps using SPM (Wellcome Department of Imaging Neuroscience, UCL, London, UK). Images subtracted pair-wise (labelled images subtracted from control images) were generated and averaged along the temporal axis. These subtracted ASL data contained signals from labelled blood in capillaries (i.e. the tissue signal) and the labelled blood in arteries, referred to as the “macrovascular” signal. Crushed subtracted data were used for the evaluation of the tissue signal. Non-crushed data minus crushed data were calculated to derive the macrovascular signals. To remove noisy pixels, the calculated results were first thresholded by the peak value of each signal computed by: (maximum (signal) > 0.035*maximum (signal_max_)). The signal_max_ was the macrovascular signal with the maximum peak. Next, the raw macrovascular signal was fitted to a modified Hrabe-Lewis model which takes the temporal dispersion effect into account [19]. Due to the long duration of the labelling pulses in pCASL, the labelled blood may have already started to reach some tissue before imaging begins. This length of 1 s for the labelling period was taken into account on the time axis of the fittings considering the leading and trailing edges of the labelled bolus [19]. In this study, a one-compartment Buxton model was used in fitting the tissue data [20] in a voxel-by-voxel manner. For each brain voxel the previously fitted macrovascular signal of the nearest arterial voxel was used as local AIF. To provide CBF in absolute units, M_0_ was estimated from the data by fitting a saturation recovery curve voxel-wise to the control images in the dataset [10]. The tissue M_0_ was used to estimate the arterial M_0_ (M_0a_) using the relationship M_0a_ = M_0_/λ, where λ is the equilibrium tissue/blood partition coefficient of water and considered to be 0.9 [21]. All fitting procedures used the nonlinear least squares algorithm (lsqnonlin) provided by Matlab (2009a). T1-weighted structural images were also measured for each subject and segmented using SPM. The grey matter (GM) and white matter (WM) were transformed into the same space as the ASL images. GM and WM masks were generated using thresholds of 0.8 and 0.9, respectively. The masks were applied to the estimated CBF images within which mean estimated CBF and standard deviation estimates were calculated.

## Results

**Fig 2:**
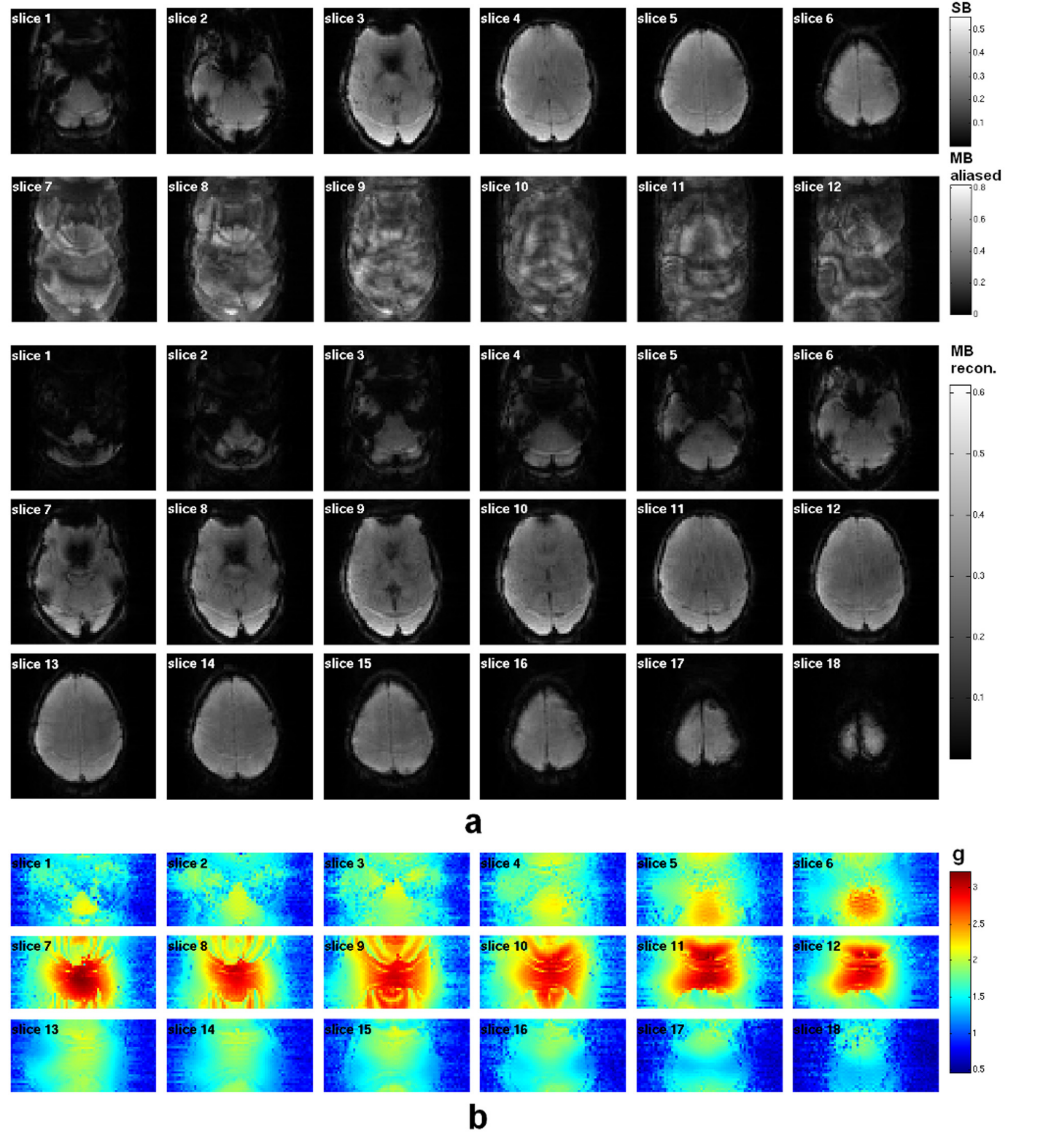
(a) EPI-based images acquired with single and multiband excitations. All the images were reconstructed after the unfolding procedure for the 2-fold in-plane parallel imaging. With the use of the MB technique, the number of slices was increased to 18 (cf. 6 in the SB data). Using MB excitation with an MB factor of 3 and blipped-CAIPI, 3 simultaneously excited slices were folded together with a phase shift. After reconstruction with the 'Slice-GRAPPA' algorithm, these 3 folded slices were clearly separated. (b) Corresponding g-factor maps from the MB technique.

For comparison, Fig 2A shows the EPI-based images acquired with the SB and MB techniques; a representative reconstructed volume is presented from each data set obtained at the very same temporal index. All images were reconstructed after the unfolding procedure was applied to account for the 2-fold in-plane acceleration introduced using parallel imaging. Three simultaneously-excited slices are aliased to be contained in one slice with the use of the MB excitation. After the ‘Slice/GRAPPA’ reconstruction, the three simultaneously-acquired slices were successfully separated from the MB-aliased images. The SNR values (0.65×mean/std) of the reconstructed images at the same temporal index are 152.5 and 119.2 for SB and MB, respectively. The g-factor map induced from the MB acceleration, presented in Fig 2B, has an averaged value of 1.4.

**Fig 3:**
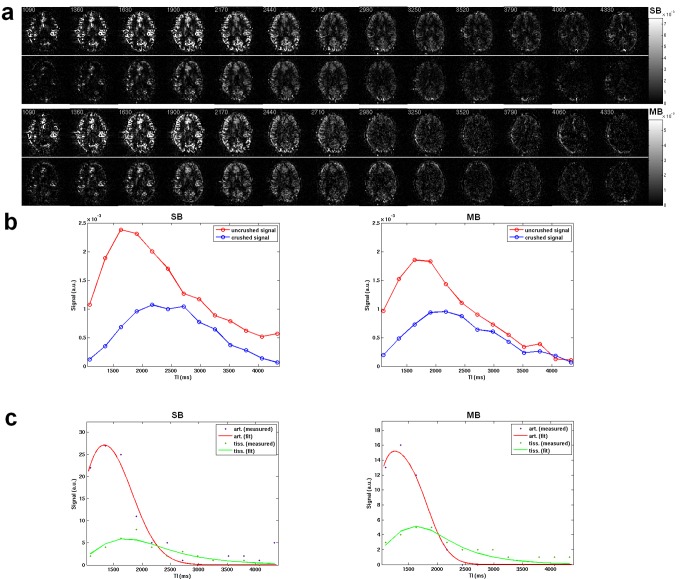
(a) Multi time-point perfusion-weighted images without crusher gradients (top row) and with crusher gradients (bottom row) with SB and MB, respectively. (b) Plot of the averaged signals from the whole brain over the time TI in ms. (c) Signal fitting of the arterial and tissue signals to the kinetic models.

Both non-crushed and crushed perfusion-weighted time series from the MB and SB methods are presented in Fig 3A. The averaged perfusion signals of the whole brain (Fig 3B) show a signal drop in the non-crushed data (-20.8%) but a comparable signal level in the crushed data (-13.4%) when using MB acceleration. The fitted kinetic curves from a voxel in the region of the temporal lobe are shown in Fig 3C. Both methods give a tight fitting of arterial signal and tissue signal with R-squared values of 0.95 and 0.85 for SB, R-square of 0.97 and 0.86 for MB.

**Fig 4:**
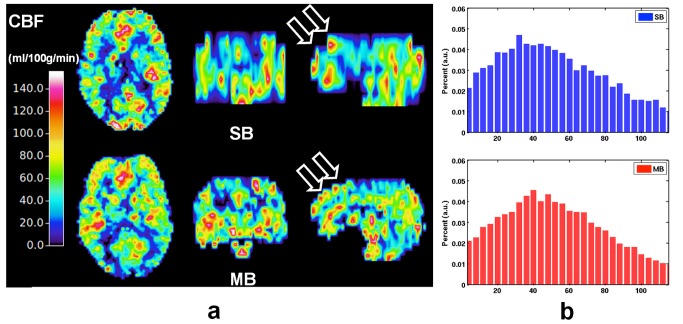
(a) Quantitative CBF ranging from 0 to 140 mL/100g/min when using the SB and MB techniques. Increased brain coverage in sagittal orientation (arrows) was observed with the MB method. (b) Corresponding histograms of the CBF from SB (top) and MB (bottom).

After fitting to the kinetic model for the whole-brain tissue signal, quantitative perfusion maps derived from one subject are shown in the 3 orthogonal views in Fig 4. Both the SB and MB-CBF fit well within the colour scale ranging from 0 to 140 mL/100g/min (Fig 4A). The enhancement resulting from the MB technique with regard to brain coverage is especially evident in the sagittal orientation. The histograms of the whole-brain CBF from the same subject (Fig 4B) show similar Gaussian-like distributions with a mean value of 56.9 mL/100g/min for SB-CBF and 54.2 mL/100g/min for MB-CBF. Furthermore, a smoother histogram of the MB-CBF can be observed when compared to the one acquired using the SB method.

**Fig 5:**
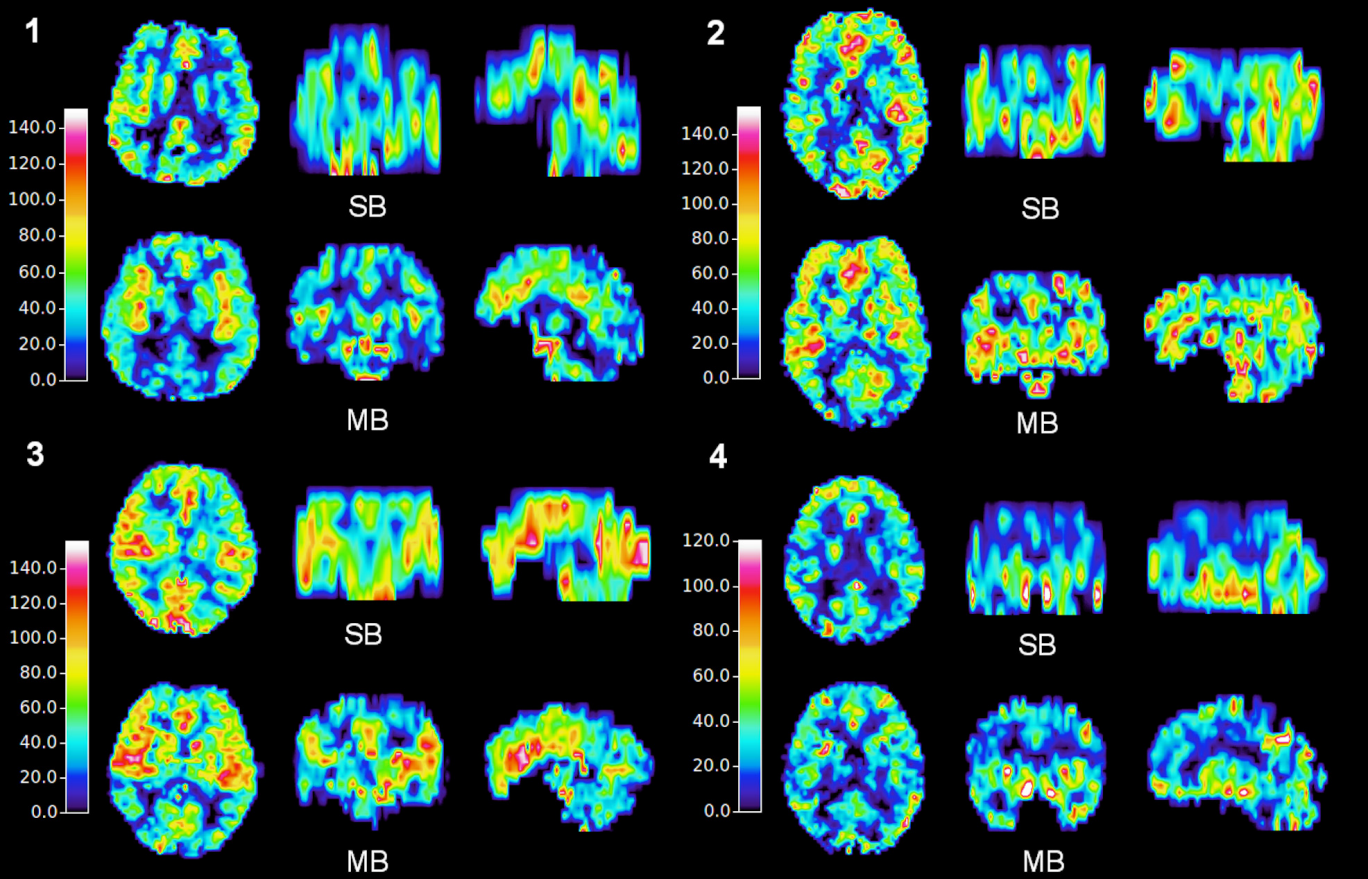
CBF maps acquired employing SB and MB methods on four subjects.

**Fig 6:**
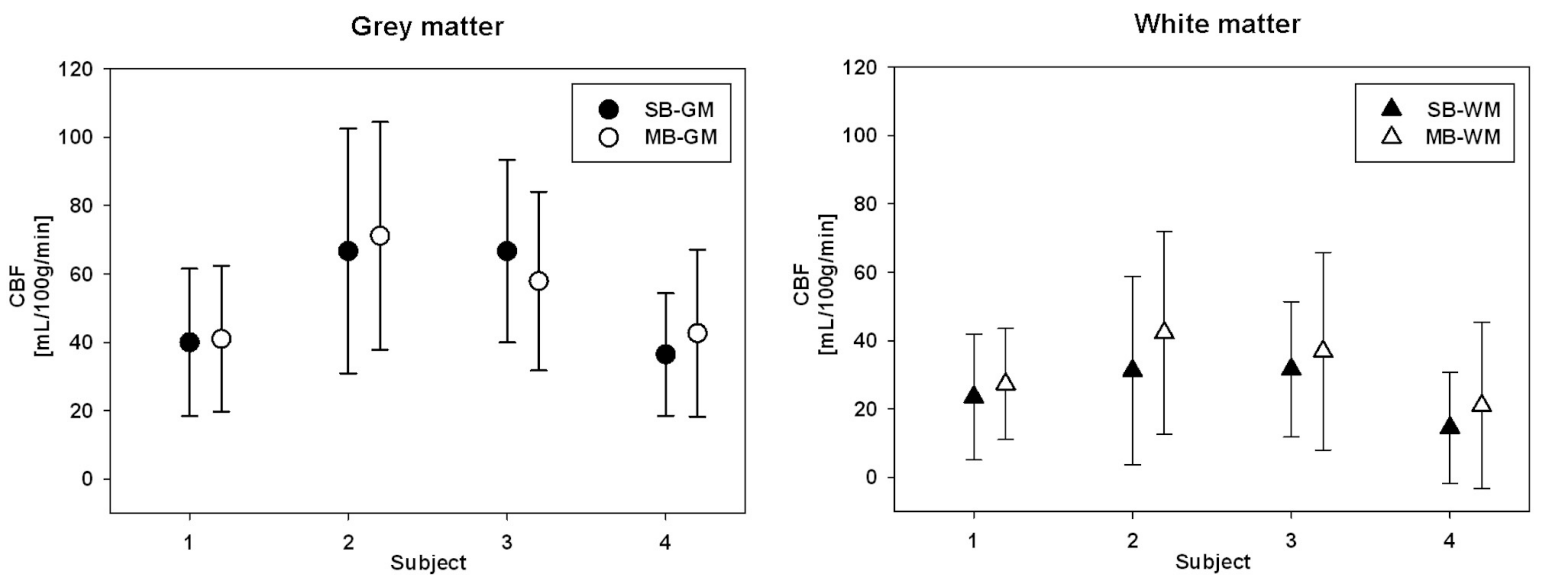
Averaged CBFs of grey matter and white matter with the SB and MB methods with respect to the subject number. The error bar indicates the standard deviation.

Whole-brain CBF maps from all four subjects are shown in Fig 5. Similar perfusion contrast was found in the SB and MB data. Discrepancy in the transverse orientation was only found due to the different slice positioning of each measurement. However, perfusion information with increased slice coverage was obtained using MB techniques. The averaged CBF values in GM were 52.5 and 53.2 mL/100g/min for SB and MB, respectively. The respective CBF values in WM were 25.2 and 31.9 mL/100g/min. The mean of the differences between the two methods, as measured across the subject, was negligible; the respective values (see Fig 6) were: GM (mean of +0.7 mL/100g/min) and WM (mean of +6.7 mL/100g/min). The differences between SB and MB data at the overlapped slices are presented in the supplementary material. Comparison of both structural (S1 Fig) and perfusion images (S2 Fig) were performed on one representative subject. Ghost and motion artefacts from MB can be found in the structural images. The perfusion maps from the MB method show an averaged increase in the WM region, which is consistent with the result across the subjects (Fig 6).

## Discussion

In this work, the MB technique was integrated with multi time-point pCASL to increase the number of slices in order to achieve whole-brain perfusion measurement. With an MB factor of 3, LL-EPI using the MB technique achieves three times the number of slices whilst maintaining the same acquisition time (6 minutes) as the SB technique. By combining MB with blipped-CAIPI, g-factor penalties in the reconstructed images can be reduced. Comparable CBF can be acquired with higher resolution after the application of MB technique.

In previous work with LL-EPI and multi time-point pulsed ASL, only a few slices could be acquired for the kinetic behaviour of blood perfusion. During the signal decay of labelled spins, multiple repetitions at different inversion times (TI) can be efficiently sampled with LL-EPI. The typical numbers of TI varied from 10~18 and the typical numbers of slices were 4~7 [7, 10, 22–24]. In order to capture the signal before it decayed entirely, the number of slices and TIs were somewhat compromised. Due to the trade-off between temporal resolution and number of slices, the temporal resolution needs to be reduced if more slices per readout are to be acquired. Multiple concatenations of the slices enable the acquisition of more slices. However, such approaches increase the total acquisition time. Rather than increasing the measurement time, an increase in the number of slices was achieved in this work using the MB technique with the same acquisition time as the conventional SB method.

The quality of the images resulting from MB acceleration is comparable to those from the SB method as shown in Fig 2A. However, the off-resonance fat signals differ between images due to the omission of a fat saturation pulse. There are no signs of visible aliasing or other artefacts. A fat saturation pulse can eliminate the fat signal but would also cause a signal drop in the perfusion weighting. For that reason, a fat saturation pulse was not included in the imaging sequences. Although, the omission of the fat saturation pulse resulted in a certain amount of ghost artefacts (see Fig 2A), the images were reconstructed without loss of spatial resolution or any severe distortions. The blipped-CAIPI method demonstrated a reduction the g-factor penalty.

According to the principles of MB acceleration [25], the impact on SNR can be described as *SNR* = *SNR*_*0*_ *∙g* ^*-1*^ where *g* is the g-factor. The SNR loss is only associated with the g-factor due to the preservation of slice signals when MB acceleration is applied [14]. There is no SNR loss due to reduced data collection along slice direction. An SNR drop by a factor of 1.3 for MB (119.2 vs. 152.5) is close to the calculated mean g-factor of 1.4 from the MB technique. The comparable SNR in the EPI-based images from both the methods guarantees similar perfusion weightings when the subsequent subtractions of control-label pairs were performed.

The resultant perfusion time series shows comparable contrasts in both the non-crushed and crushed data between the SB and MB readout (Fig 3A). As indicated by the perfusion-weighted time series from MB and SB in Fig 3B, the signal drop was 20.8% in the non-crushed data and 13.4% in the crushed data after using MB acceleration. These values were calculated based on the maximum of the signal in time. With a comparable signal level in the perfusion time series, successful fitting of the non-crushed and crushed data was always possible (cf. Fig 3C).

Alongside perfusion contrast which is similar, CBF data with more extended brain coverage was achieved (Figs 4A and 5) following MB acceleration. Except for the increased slice and voxel number, a similar histogram was obtained with MB-CBF (Fig 4A).

By combining the non-crushed and crushed data, arterial and tissue perfusion information can be acquired with one measurement. To suppress contamination of the signal from macrovascular vessel contributions, a crusher gradient of 8 cm/s was applied in 7 cycles with different directions. The advantage of cycled flow suppression is that it can fully suppress macrovascular flow in all directions and provide tissue-only data. Further use of stronger suppression gradients to suppress all flowing blood and acquire a pure extravascular signal [26] can also be considered.

When compared to standard SB-CBF, MB-CBF shows more difference in WM (+6.7 mL/100g/min) than GM (+0.7 mL/100g/min) as presented in Fig 6. WM-CBF with the MB technique shows an increased value. The reason for this might be due to the lower SNR in WM and the number of AIFs after using the MB method (Fig 3). Due to the longer BAT of WM, the signal decays more rapidly leading to a lower SNR. An even lower SNR results from the application of the MB method. With the MB method, the number of local AIFs is increased, thereby providing a more reliable estimate of regional CBF.

The averaged GM-CBF from 4 subjects is approximately 53 mL/100g/min and consequently is also within the range of PET results, as reported in a previous MR-PET study [27] using single-shot pCASL. From that study, where 10 young healthy subjects were evaluated, GM-CBF was 67.3 mL/100g/min and WM-CBF was 19.5 mL/100g/min. With PET, the comparable values of GM-CBF were 51.8 mL/100g/min and WM-CBF of 17.4 mL/100g/min. A further standard reference is Leenders et al., who published a mean GM-CBF of 54.8±12.0 mL/100g/min with the ^15^O steady-state inhalation method (PET), in 34 healthy volunteers [28]. To evaluate the true CBF in GM using the current technique, it is necessary to measure a larger number of subjects.

A model-based analysis using analytical functions was used to fit the CBF, which takes the bolus dispersion into account. Another option, namely model-free fitting, using a deconvolution method similar to dynamic susceptibility contrast perfusion measurements could be also applied. The numerical deconvolution required in a model-free analysis is typically performed using singular value decomposition of the discrete convolution matrix [29]. This deconvolution method is known to underestimate the true CBF as well as introducing physiological oscillations in the residual function [7, 10]. The analysis method can still be optimized by using Bayesian nonlinear model fitting [10] or other models [30].

To minimize T_2_* decay in the EPI-based readout and also to maximize T_1_ contrast in the images, a short TE is required, which can be realised by reducing the number of phase encode lines. In-plane parallel imaging with an iPAT factor of 2 was used in our work for this purpose. Partial Fourier and multi-shot are other candidate methods to reduce the TE. Fat saturation was not performed due to the risk of off-resonance artefacts in the raw data. Further improvement could be obtained using water-selective excitation RF pulses. The acceleration factor of the MB technique in the current application with a 32-channel coil was three. Higher MB factors (e.g. 5) could be used in the future by adjusting the peak voltage of RF pulse [15] and consequentially the SNR lost in perfusion signals. The aim of this study was to prove the feasibility and advantages of the MB technique in the LL readout of multi time-point pCASL. Multi time-point ASL can also be achieved by Hadamard time-encoded labelling approaches [24, 31,32].

## Conclusions

This study demonstrates quantitative perfusion measurement acquired with MEM-pCASL using an MB accelerated LL-EPI readout. With the addition of the MB technique, whole-brain CBF can be acquired with three times the number of slices within the same measurement time when compared with the SB method.

## Supporting Information

S1 Fig Comparison of structural single-band and multiband images at the overlapped slices. The top and middle rows show EPI-based structural images acquired with multiband and single-band excitations, respectively; the images in each column were obtained at an identical slice position. The bottom row shows the differences between the top row and middle row images.
(DOC)

S2 Fig Comparison of perfusion images acquired by single-band and multiband methods. The top and middle rows show perfusion maps acquired with multiband and single band excitations, respectively, where the range is 0 ~ 150 mL/100g/min; the images in each column were obtained at an identical slice position. The bottom row shows the differences between the top row and middle row images, where the range is -50 ~ 50 mL/100g/min.
(DOCX)
