# Supplementary material for: Tripled Readout Slices in Multi Time-Point pCASL Using Multiband Look-Locker EPI
Source: PLoS One. 2015 Nov 6;10(11):e0141108. doi: 10.1371/journal.pone.0141108 (PMC4636240; doi:10.1371/journal.pone.0141108)
Supplement: S1 Fig — The top and middle rows show EPI-based structural images acquired with multiband and single-band excitations, respectively; the images in each column were obtained at an identical slice position. The bottom row shows the differences between the top row and middle row images. (DOC) [file pone.0141108.s001.doc]

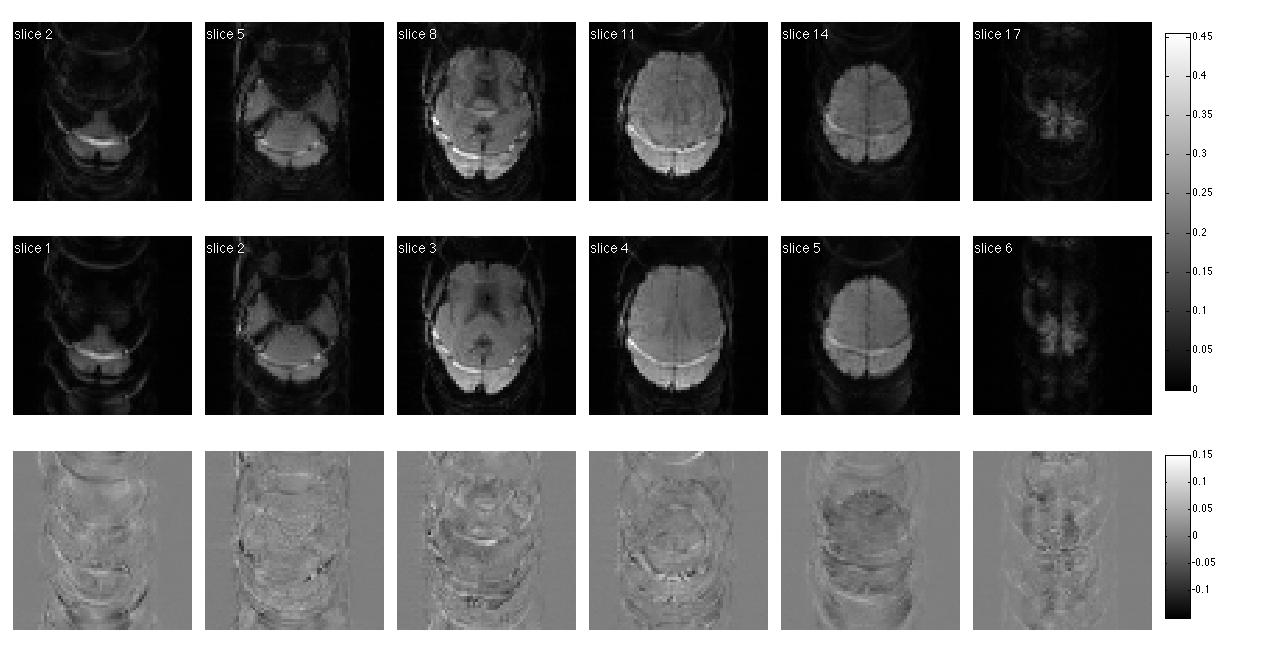


**S1 Fig. Comparison of structural single-band and multiband images at the overlapped slices.** The top and middle rows show EPI-based structural images acquired with multiband and single-band excitations, respectively; the images in each column were obtained at an identical slice position. The bottom row shows the differences between the top row and middle row images.
