# Supplementary material for: Tripled Readout Slices in Multi Time-Point pCASL Using Multiband Look-Locker EPI
Source: PLoS One. 2015 Nov 6;10(11):e0141108. doi: 10.1371/journal.pone.0141108 (PMC4636240; doi:10.1371/journal.pone.0141108)
Supplement: S2 Fig — The top and middle rows show perfusion maps acquired with multiband and single band excitations, respectively, where the range is 0 ~ 150 mL/100g/min; the images in each column were obtained at an identical slice position. The bottom row shows the differences between the top row and middle row images, where the range is -50 ~ 50 mL/100g/min. (DOCX) [file pone.0141108.s002.docx]

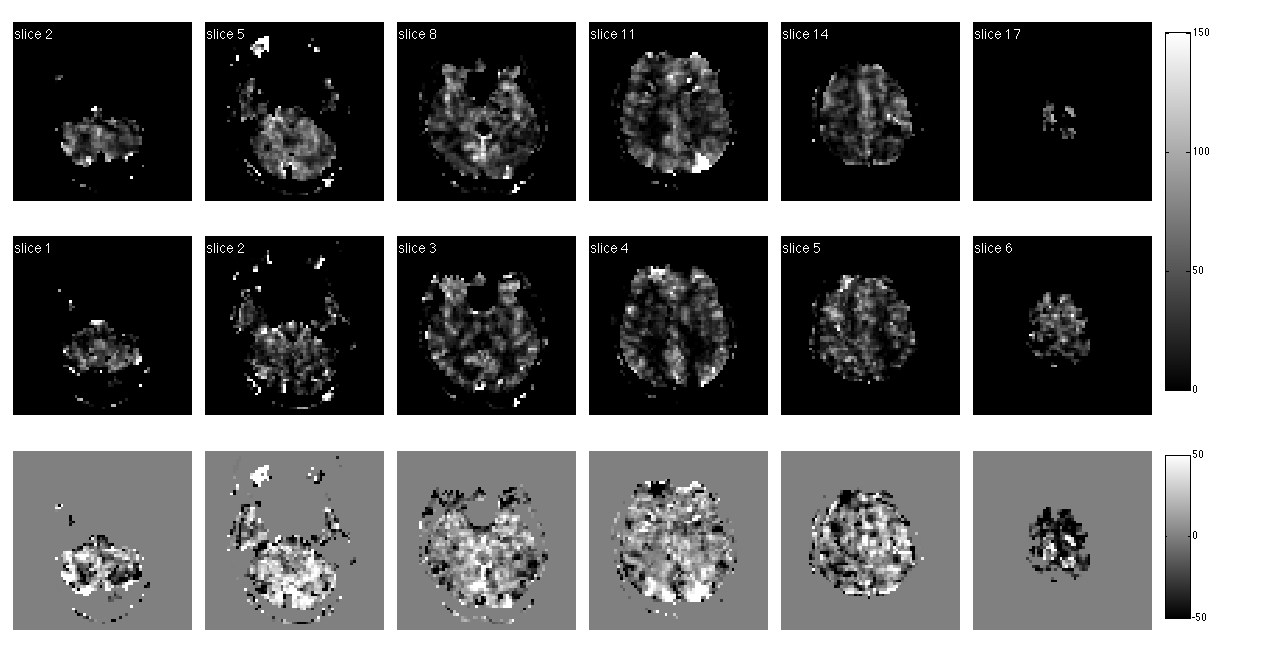
**.**

S2 Fig. **Comparison of perfusion images acquired by single-band and multiband methods.** The top and middle rows show perfusion maps acquired with multiband and single band excitations, respectively, where the range is 0 ~ 150 mL/100g/min; the images in each column were obtained at an identical slice position. The bottom row shows the differences between the top row and middle row images, where the range is -50 ~ 50 mL/100g/min.
